# Pigs with an *INS* point mutation derived from zygotes electroporated with CRISPR/Cas9 and ssODN

**DOI:** 10.3389/fcell.2023.884340

**Published:** 2023-01-13

**Authors:** Fuminori Tanihara, Maki Hirata, Zhao Namula, Lanh Thi Kim Do, Naoaki Yoshimura, Qingyi Lin, Koki Takebayashi, Tetsushi Sakuma, Takashi Yamamoto, Takeshige Otoi

**Affiliations:** ^1^ Faculty of Bioscience and Bioindustry, Tokushima University, Tokushima, Japan; ^2^ Bio-Innovation Research Center, Tokushima University, Tokushima, Japan; ^3^ College of Coastal Agricultural Sciences, Guangdong Ocean University, Zhanjiang, Guangdong, China; ^4^ Faculty of Veterinary Medicine, Vietnam National University of Agriculture, Hanoi, Vietnam; ^5^ Graduate School of Integrated Sciences for Life, Hiroshima University, Hiroshima, Japan

**Keywords:** CRISPR/Cas9, INS, *in vitro* fertilized zygotes, pig, point mutation, ssODN

## Abstract

Just one amino acid at the carboxy-terminus of the B chain distinguishes human insulin from porcine insulin. By introducing a precise point mutation into the porcine *insulin* (*INS*) gene, we were able to generate genetically modified pigs that secreted human insulin; these pigs may be suitable donors for islet xenotransplantation. The electroporation of the CRISPR/Cas9 gene-editing system into zygotes is frequently used to establish genetically modified rodents, as it requires less time and no micromanipulation. However, electroporation has not been used to generate point-mutated pigs yet. In the present study, we introduced a point mutation into porcine zygotes *via* electroporation using the CRISPR/Cas9 system to generate *INS* point-mutated pigs as suitable islet donors. We first optimized the efficiency of introducing point mutations by evaluating the effect of Scr7 and the homology arm length of ssODN on improving homology-directed repair-mediated gene modification. Subsequently, we prepared electroporated zygotes under optimized conditions and transferred them to recipient gilts. Two recipients became pregnant and delivered five piglets. Three of the five piglets carried only the biallelic frame-shift mutation in the *INS* gene, whereas the other two successfully carried the desired point mutation. One of the two pigs mated with a WT boar, and this desired point mutation was successfully inherited in the next F1 generation. In conclusion, we successfully established genetically engineered pigs with the desired point mutation *via* electroporation-mediated introduction of the CRISPR/Cas9 system into zygotes, thereby avoiding the time-consuming and complicated micromanipulation method.

## 1 Introduction

Diabetes mellitus is a major contributor to public health issues, and the number of patients with diabetes is increasing globally (Shaw et al., 2010). The pathogenesis of type 1 diabetes is caused by the immunological destruction of islet *β* cells producing insulin. Thus, pancreatic islet transplantation, without the need for insulin injections, is considered an effective treatment strategy (Atkinson and Eisenbarth, 2001). However, islet transplantation is limited due to the lack of islet donors (Frank et al., 2005).

The anatomical and physiological properties of pigs are similar to humans. Therefore, genetically modified pigs are expected to be ideal organ donors for xenotransplantation. Islets collected from pigs are an attractive alternative source for islet xenotransplantation. Thus, studies have been conducted on the microencapsulation of islets (Basta et al., 2011; Hillberg et al., 2013) and genetic modifications (Kemter et al., 2018) to protect porcine islets from the host immune system. Porcine insulin differs from human insulin by one amino acid at the carboxy-terminus of the B chain (alanine in pigs and threonine in humans) (Sonnenberg and Berger, 1983). The conversion of a single nucleotide at the given position of the porcine *insulin* (*INS*) gene (introduction of a point mutation), which changes G to an A at codon 54 (GCC, encoding alanine, to ACC, encoding threonine), converts porcine insulin to human insulin and enables the generation of genetically engineered pigs secreting human insulin (Yang et al., 2016), thereby establishing a suitable donor for the xenotransplantation of islets.

Gene editors—such as clustered regularly interspaced short palindromic repeats-associated protein 9 (CRISPR-Cas9) nucleases (Cong et al., 2013; Mali et al., 2013)—have enabled the insertion of precise base mutations into the genomic DNA of cells, including zygotes and embryonic cells (Inui et al., 2014; Yin et al., 2014; Yamamoto and Gerbi, 2018). After gene editors induce double-strand breaks (DSBs) in DNA, non-homologous end-joining (NHEJ) or homology-directed repair (HDR) pathways act to repair the DNA (Kanaar et al., 1998). The NHEJ repair pathway can induce random insertions or deletions (indels) and disrupt the functions of targeted genes. By contrast, the precise repair of DNA after DSBs is achieved *via* the HDR pathway using donor DNA that has a homologous region from sister chromatids, homologous chromosomes, or exogenous DNA. CRISPR/Cas9-mediated HDR can be used to introduce a point (or small) mutation *via* the simultaneous introduction of a single-stranded oligodeoxynucleotide (ssODN), which has a right and left arm homologous with the target region, as the donor DNA encoding the desired point mutation (Inui et al., 2014).

In pigs, point mutations have either been introduced *via* the microinjection of gene editors with ssODN into zygotes/embryos (Zhou et al., 2016) or a somatic cell nuclear transfer (SCNT) technique using gene-edited somatic cells (Yang et al., 2016; Montag et al., 2018; Li et al., 2020). Previously, we established the GEEP (gene editing by electroporation of Cas9 protein) method, which can successfully introduce the CRISPR/Cas9 system into porcine zygotes *via* electroporation, resulting in highly efficient disruption of the targeted gene (Tanihara et al., 2016). GEEP requires considerably less time with no need for advanced skills for micromanipulation. In mice, electroporation is widely used to transfer exogenous molecules for genome engineering, and generating knock-in (Miyasaka et al., 2018) and point mutations (Kim et al., 2017; Teixeira et al., 2018). In pigs, we previously succeeded in introducing a precise point mutation into porcine zygotes *via* electroporation (Wittayarat et al., 2021). However, there have been no reports concerning the generation of point mutated pigs *via* electroporation.

Since most DSBs generated by Cas9 are subjected to the NHEJ pathway, the efficiency of CRISPR/Cas9-mediated HDR is inherently low (Frit et al., 2014; Liu et al., 2018). In SCNT, the low efficiency of HDR is manageable by selecting somatic cells carrying the desired mutations. However, improvements in HDR efficiency are crucial for the one-step introduction of HDR-mediated mutations into zygotes *via* microinjection and electroporation. DNA ligase IV is a key enzyme of the NHEJ pathway and 5,6-Bis-(benzylideneamino)-2-mercaptopyrimidin-4-ol (Scr7) is a DNA ligase IV inhibitor. Therefore, supplementation with Scr7 improves HDR efficiency by inhibiting the NHEJ pathway (Srivastava et al., 2012; Maruyama et al., 2015). Furthermore, HDR efficiency is affected by the homology arm length of the ssODN donor in porcine somatic cells (Wang et al., 2016) and zygotes (Wittayarat et al., 2021). It is also crucial to optimize the ssODN length for efficient HDR before embryo transfer. In the present study, we optimized the HDR-mediated introduction of a precise point mutation into the *INS* target region of porcine zygotes using the GEEP method and generated pigs with *INS* point mutations.

## 2 Materials and methods

### 2.1 Animals

All animal care and experimental procedures were performed according to the guidelines for animal experiments of Tokushima University and the ARRIVE guidelines. Animal husbandry and anesthesia/euthanasia procedures were carried out as described previously (Tanihara et al., 2020a). The Prefectural Livestock Research Institute (Tokushima, Japan) provided two sexually mature Landrace gilts. Humane endpoints were defined as refusal of food or drink, symptoms of suffering, or decreased body weight resulting from the *INS* modification. In the present study, euthanasia was performed on a few *INS-*mutant piglets showing signs of poor general health conditions and extremely high blood glucose levels.

### 2.2 Oocyte collection, *in-vitro* maturation (IVM), and *in-vitro* fertilization (IVF)

Oocyte collection, IVM, and IVF were performed as described previously (Nguyen et al., 2017). Pig ovaries were collected at a local slaughterhouse from prepubertal gilts. We collected cumulus–oocyte complexes and cultured them in a maturation medium for 44 h. The matured oocytes were co-incubated with frozen–thawed ejaculated spermatozoa (1 × 10^6^ cells/ml) for 5 h in a porcine fertilization medium (Research Institute for the Functional Peptides Co., Yamagata, Japan), and subsequently cultured in porcine zygote medium (PZM-5; Research Institute for the Functional Peptides Co.) for 7 h prior to gene editing using electroporation. The oocytes were then incubated in a humidified incubator at 39°C and 5% CO_2_.

### 2.3 Design of gRNA sequence

Alt-R CRISPR crRNAs and the tracrRNA system, supplied by Integrated DNA Technologies (IDT; Coralville, IA, United States), were used for guide RNA (gRNA). The CRISPR direct web tool (https://crispr.dbcls.jp/) was used to design the gRNA sequence (Naito et al., 2015). To minimize off-target effects, the COSMID web tool (https://crispr.bme.gatech.edu/) (Cradick et al., 2014) was used to confirm that the 14 nucleotides at the 3′end of the designed gRNAs matched the target regions of *INS* genes.

### 2.4 Electroporation and *in-vitro* culture

Electroporation with ssODN was performed as described previously (Wittayarat et al., 2021). The fertilized zygotes were placed in a line in the electrode gap in a chamber slide (LF501PT1-20; BEX, Tokyo, Japan) filled with Nuclease-Free Duplex Buffer (IDT) containing 100 ng/μl gRNA targeting the porcine *INS* gene, 100 ng/μl Cas9 protein (Guide-it Recombinant Cas9; Takara Bio, Inc., Shiga, Japan), and 16 pmol/μl ssODN. Thereafter, zygotes were electroporated (five 1-ms pulses at 25 V) using a CUY21EDIT II electroporator (BEX). After electroporation, to examine the genotypes of the resulting blastocysts along with the zygotes’ competence to develop into the blastocyst stage, zygotes were cultured either for 12 h in PZM-5 until embryo transfer or for 3 days in PZM-5, followed by 4 days in porcine blastocyst medium (Research Institute for the Functional Peptides Co.). Zygotes and embryos were incubated in a humidified incubator at 39°C with 5% CO_2_, 5% O_2_, and 90% N_2_.

### 2.5 Analysis of targeted gene sequence after electroporation

Genomic DNA was isolated from individually collected blastocysts, and the genomic regions flanking the gRNA target site were PCR-amplified using KOD One PCR master mix (Toyobo, Osaka, Japan) and the specific primers: 5′- AGG​ACG​TGG​GCT​CCT​CTC​TC-3′ (forward) and 5′- GGG​CCT​TGA​CTC​CGT​AAG​AT-3′ (reverse). The PCR products were extracted using agarose gel electrophoresis, and the genotype of each blastocyst was analyzed using Sanger sequencing followed by application of the TIDE (tracking of indels by decomposition) bioinformatics package (Brinkman et al., 2014).

### 2.6 Embryo transfer

The preparation of recipient gilts for embryo transfer was performed as described previously (Onishi et al., 2000). Four to 7 weeks after mating, pregnant gilts were administered .2 mg of cloprostenol (Planate; MSD Animal Health, Tokyo, Japan) *via* intramuscular (i.m.) injection. After 24 h, the gilts were administered a second i.m. injection of .2 mg cloprostenol and 1000 IU eCG (PMSA for Animal, ZENOAQ, Fukushima, Japan). Then, 72 h after the eCG injection, recipient gilts were administered an i.m. injection of 1500 IU hCG (Gestron 1500, Kyoritsu Seiyaku) to induce the estrus. Subsequently, 100 embryos electroporated 12 h before embryo transfer were transferred into each oviduct of a recipient gilt 72 h after the hCG i.m. injection, resulting in the transfer of 200 embryos per gilt.

### 2.7 Mutation analysis in blastocysts and piglets using deep sequencing

Individually collected blastocysts and tissue samples were used to isolate genomic DNA. Following the manufacturer’s instructions, two-step PCR using specific primers and index PCR primers was performed to amplify the genomic regions flanking the gRNA target site (Illumina, Hayward, CA, United States; Supplementary Table S1). The amplicons were submitted to MiSeq sequencing using the MiSeq Reagent Kit v. 2 after gel purification (250 cycles; Illumina). CRISPResso2 (Clement et al., 2019) was used for data analysis. To minimize false-positive classification, indels were assessed within a five base pair (bp) window surrounding the predicted cleavage site (Gaj et al., 2017). Genomic DNA isolated from wild-type (WT) pig-derived ear biopsies was used as the control. Sequencing errors were defined as a small number of amplicons carrying different sequences that were also observed in the control sample.

### 2.8 Off-target analysis using deep sequencing

An off-target analysis was performed as described previously (Tanihara et al., 2016). Genomic DNA of delivered pigs derived from electroporated zygotes and of two control WT pigs were individually used as templates for PCR. Potential off-target sites were chosen using the COSMID webtool (Cradick et al., 2014), which ranks off-target sites based on the number and position of mismatches. The six top-ranked potential off-target sites were analyzed using deep sequencing with specific primers and index PCR primers (Supplementary Table S2), as described in Section 2.7.

### 2.9 Experimental design

#### 2.9.1 Experiment 1: Effect of Scr7 concentration on point mutation introduction efficiency

**FIGURE 1:**
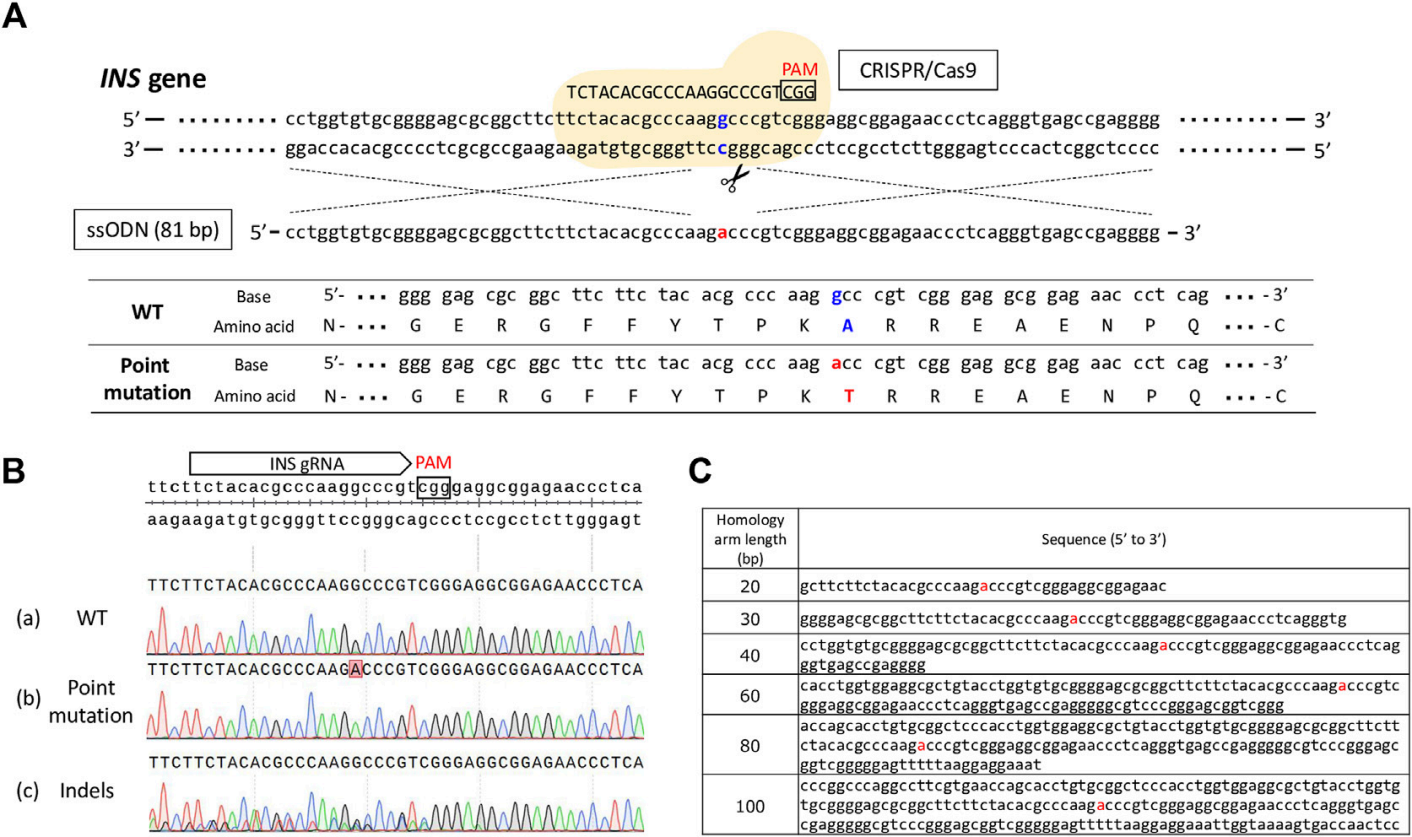
**(A)** Schematic of the experimental design to introduce a desired point mutation into the porcine *INS* gene. Electroporation was used to deliver the Cas9 protein, gRNA targeting the *INS* gene, and single-stranded oligodeoxynucleotide (ssODN) to porcine zygotes. The sequence of ssODN with 40 bp homology arms is presented in this illustration. The flanking amino acid sequences of the target site before and after the introduction of the point mutation are also presented. Mutated nucleotides and converted amino acids are indicated in red. The original nucleotide sequence in porcine *INS* gene and original amino acid sequence before the introduction of a point mutation are indicated in blue. PAM, protospacer adjacent motif sequence; WT, wild-type. **(B)** Representative results of Sanger sequencing of the blastocysts derived from electroporated zygotes with the Cas9 protein, *INS* gRNA, and ssODN. (a) Wild-type (WT) blastocyst. (b) Point-mutated blastocyst. (c) Blastocyst carrying indels introduced by the Cas9 protein and gRNA. **(C)** Sequences of the ssODN having various homology-arm lengths.

First, we designed gRNAs targeting porcine *INS* (target sequence: 5′-TCT​ACA​CGC​CCA​AGG​CCC​GT-3′) and ssODN carrying right and left 40-bp homology arms as homology donors to optimize the efficiency of the HDR-mediated introduction of a precise point mutation. A mixture of gRNA targeting the porcine *INS* gene, Cas9 protein, ssODN, and Scr7 (Xcessbio Biosciences, Inc., San Diego, CA, United States) was used to induce single amino acid conversion *via* electroporation in porcine zygotes. As a target, we attempted to change GCC, encoding alanine, at codon 54 to ACC, encoding threonine (Figure 1A). To evaluate the efficiency at which Scr7 introduced a point mutation, we electroporated gRNA, Cas9 protein, and ssODNs with multiple concentrations of Scr7 (.5, 1, 2, and 4 μM) or without Scr7 (0 μM) into porcine putative zygotes, 12 h after the start of IVF. After *in-vitro* culture for 7 days, the resulting blastocysts were subjected to genotype analysis, as described above. Blastocysts that carried only the WT sequence were classified as WTs. Blastocysts carrying the desired point mutation without other mutations were classified as those with a point mutation. Blastocysts that carried more than one type of mutation (insertion and/or deletion) near the gRNA-targeting site were classified as indels (Figure 1B).

#### 2.9.2 Experiment 2: Effect of homology arm length on point mutation introduction efficiency

The length of ssODNs as homology donors affects HDR efficiency (Wang et al., 2016; Wittayarat et al., 2021). We designed six ssODNs with homology arms of different lengths to optimize the efficiency of HDR-mediated point mutations (Figure 1C). We introduced Cas9 protein with gRNA and 16 pmol/μl of each ssODN into *in-vitro*-fertilized zygotes *via* electroporation. After *in-vitro* culture for 7 days, we evaluated the frequency of detected mutations in the *INS* target region of the blastocysts. Blastocyst genotypes were classified according to the criteria of embryo genotypes described in 2.9.1.

#### 2.9.3 Experiment 3: Generation and analysis of genetically modified pigs

Next, we investigated whether genetically modified pigs carrying point mutations can be generated from electroporation-mediated point-mutated zygotes. Using electroporation, we introduced the Cas9 protein, gRNA targeting the *INS* gene, ssODNs carrying the 40-bp homology arms, and 1 µM Scr7 into the zygotes. The zygotes were then transferred to the two recipients ∼12 h after electroporation or cultured until the blastocyst stage. After delivery of piglets, we carefully monitored the body condition of the piglets, and performed euthanasia based on signs of prostration. The genotypes of and off-target effects in the delivered piglets were analyzed using deep sequencing. To evaluate the gene editing outcomes accurately, the genotypes of genetically modified blastocysts were also analyzed.

Furthermore, we investigated whether the introduced point mutations were inherited by the next generation. The pig with the successful introduction of the desired point mutation was mated with a WT boar. The genotypes of the F1 piglets were analyzed using Sanger sequencing and TIDE.

### 2.10 Statistical analysis

All percentage data were subjected to arcsine transformation, then analyzed by analysis of variance followed by Fisher’s protected least significant difference test. The percentage of mutated blastocysts was analyzed using chi-squared tests with Yates’ correction. We used the StatView software (Abacus Concepts, Berkeley, CA, United States) for statistical analysis. Differences with a probability value (*p*) ≥ .05 were considered statistically significant, and those with a probability value ≥.1 were considered marginally significant.

### 2.11 Study approval

The Institutional Animal Care and Use Committee of Tokushima University approved the animal experiments conducted in the present study (approval number: T2019-11).

## 3 Results

### 3.1 Experiment 1: Effect of Scr7 concentration on point mutation introduction efficiency

**FIGURE 2:**
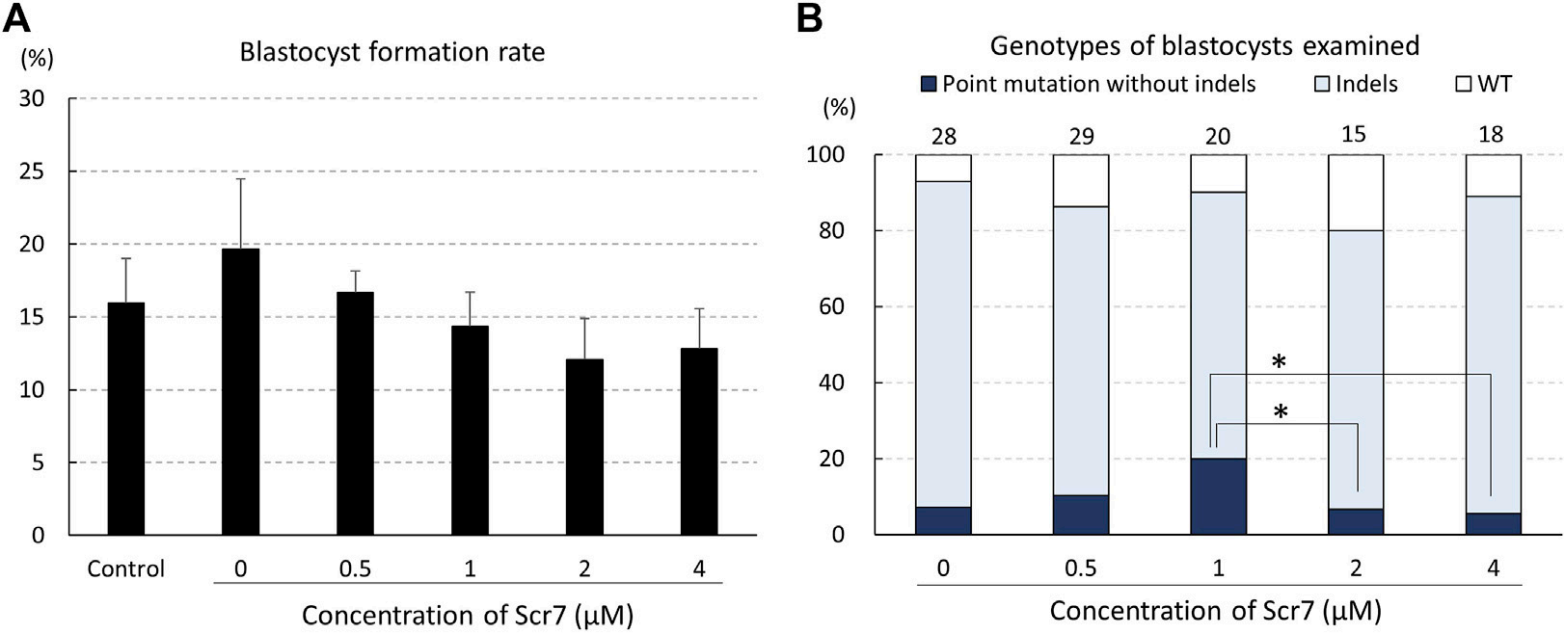
Rates of blastocyst formation **(A)** and genotypes of blastocysts **(B)** developed from zygotes electroporated with the CRISPR/Cas9 system targeting the *INS* gene and single-stranded oligodeoxynucleotide (ssODN) with various Scr7 concentrations. The percentage of point mutations and indels indicates the ratio of the number of blastocysts carrying a point mutation/indel to the total number of blastocysts examined. Five replicate trials were carried out, and numbers above the bars indicate total number of blastocysts examined. Point mutation without indels, blastocyst carrying a point mutation without another mutation; Indels, blastocyst carrying mutations around the gRNA-targeting site; WT, wild-type. Error bars indicate the mean ± SEM **(A)**. *p < .1.

The concentration of Scr7 did not have a statistically significant effect on blastocyst formation rates from electroporated zygotes (Figure 2A). Of the zygotes electroporated with ssODNs, 5.6%–20.0% developed into blastocysts carrying a point mutation (Figure 2B). The ratio of blastocysts that carried a point mutation to the total number of examined blastocysts in the group treated with 1 μM Scr7 (20.0%) was higher (*p* < .1) than that in the groups treated with 2 μM (6.7%) and 4 μM (5.6%) Scr7.

### 3.2 Experiment 2: Effect of homology arm length on point mutation introduction efficiency

**FIGURE 3:**
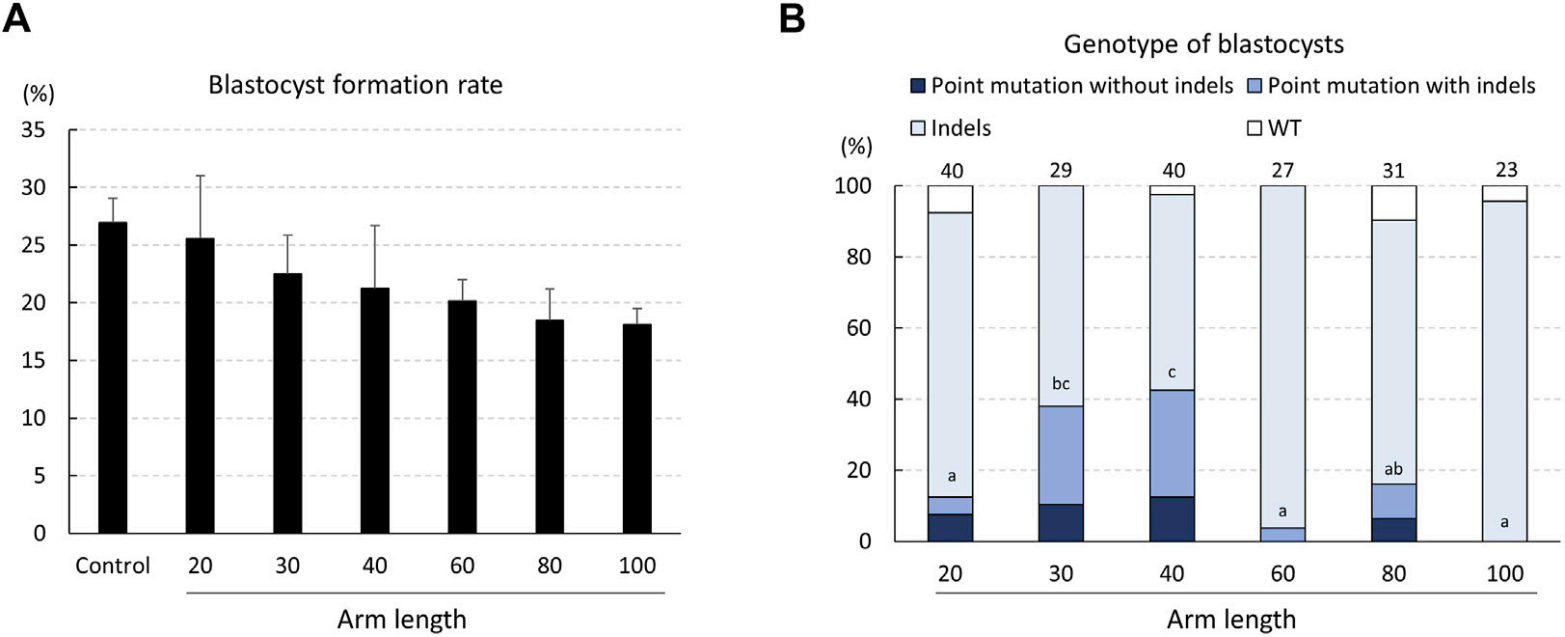
Rates of blastocyst formation **(A)** and genotypes of blastocysts **(B)** developed from zygotes electroporated with the CRISPR/Cas9 system targeting the *INS* gene and single-stranded oligodeoxynucleotide (ssODN) with different homology arm lengths. The percentages of point mutations and indels indicate the ratio of the number of blastocysts carrying a point mutation/indel to the total number of blastocysts examined. Four replicate trials were carried out and numbers above the bars indicate the total number of blastocysts examined. Point mutation without indels, blastocyst carrying a point mutation without another mutation; Point mutation with indels, blastocyst carrying a point mutation with another mutation; Indels, blastocyst carrying mutations around the gRNA-targeting site; WT, wild-type. Error bars indicate the mean ± SEM **(A)**. ^a–c^ *p* < .05.

The development of electroporated zygotes into blastocysts was statistically unaffected by the length of the ssODNs (Figure 3A). The genotypes of the blastocysts, determined using TIDE, showed that blastocysts treated with gRNA and ssODN having 20-, 30-, 40-, 60-, and 80-bp homology arms carried the desired point mutation in the *INS* target region (at rates of 12.5%, 37.9%, 42.5%, 3.7%, and 16.1%, respectively; Figure 3B). The proportion of blastocysts carrying point mutations was significantly higher (*p* < .05) in zygotes wherein ssODNs with 40-bp homology arms were introduced when compared to that with 20-, 60-, and 80-bp homology arms. Furthermore, the highest rate of blastocysts carrying point mutations without indels was observed in zygotes with ssODNs with 40-bp homology arms (12.5%).

### 3.3 Experiment 3: Generation and analysis of gene-modified pigs

**FIGURE 4:**
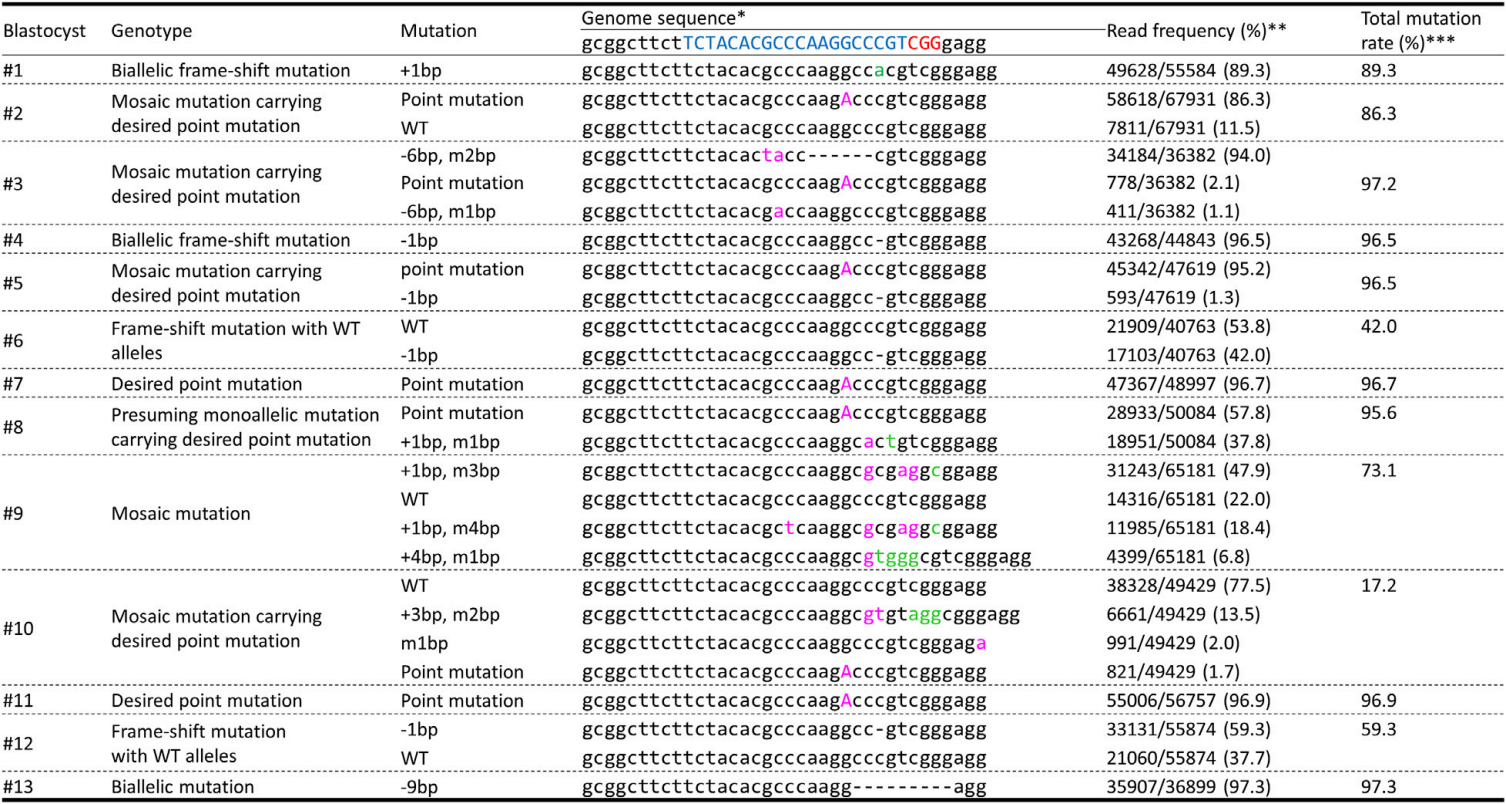
Deep-sequencing analysis of the *INS* target regions in blastocysts obtained using the same procedure adopted to generate genetically modified pigs carrying point mutations. * The target and PAM sequences of gRNA are indicated in blue and red, respectively. Inserted and modified sequences are represented by green and pink, respectively. ** The frequency was calculated by dividing the number of amplicons by the total number of reads. *** The mutation rate was calculated by dividing the total number of mutant amplicons by the total number of reads.

We electroporated gRNA, Cas9 protein, and ssODNs carrying 40-bp homology arms with 1 μM Scr7 into zygotes and the genotypes of the resulting genetically modified blastocysts were analyzed using deep sequencing to evaluate mosaicism (Figure 4). Four of thirteen blastocysts carried the mosaic mutation, including desired point mutated alleles. Two of thirteen blastocysts carried the desired point mutation biallelically.

**FIGURE 5:**
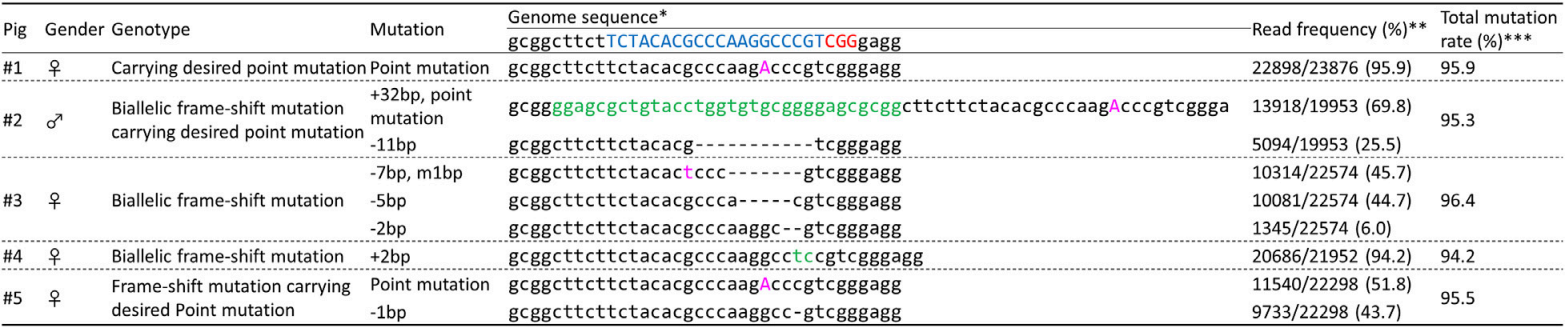
Deep-sequencing analysis of the *INS* target regions in ear biopsies from delivered piglets. * The target and PAM sequences of gRNA are indicated in blue and red, respectively. Inserted and modified sequences are represented by green and pink, respectively. ** The frequency was calculated by dividing the number of amplicons by the total number of reads. *** The mutation rate was calculated by dividing the total number of mutant amplicons by the total number of reads.

Next, we electroporated gRNA, Cas9 protein, and ssODNs carrying 40-bp homology arms with 1 μM Scr7 into zygotes and then transferred them into the oviducts of two recipient gilts. One hundred embryos were transferred into each oviduct of a recipient gilt. Both recipients became pregnant and gave birth to five piglets. Deep sequencing analysis of the target sites of *INS* genomic regions in ear biopsies revealed that all piglets carried *INS* mutations (Figure 5). Piglets #1 and #5 had the desired point mutations and showed normal growth (deep sequencing analysis using ear samples demonstrated that #1 carried only the desired point mutation; #5 carried the desired point mutation allele and a 1-bp deletion allele). In contrast, piglets #2, #3, and #4 had biallelic frameshift mutations (#2 carried a 32-bp insertion allele; #3 carried a 7-bp deletion with 1-bp modification, 5-bp deletion, and 2-bp deletion alleles; and #4 carried only a 2-bp insertion allele), which indicated knockout of the *INS* gene. Then, we performed off-target analysis using deep sequencing, thereby indicating no detected differences in six potential off-target sites without OT1 (Table 1). Off-target analysis of OT1 in pigs #1, #2, and #3 detected approximately 38%–49% of the modified sequence. However, the same modification (2-bp deletion) was detected in one of the WT genomic DNA fragments, which was considered a monoallelic single nucleotide polymorphism.

**TABLE 1:** Frequencies of the mutant sequence at potential off-target sites analyzed using deep sequencing*.

Sample	Percentages of sequences with substitutions, deletions, or insertions					
	OT1	OT2	OT3	OT4	OT5	OT6
Control 1	38.94	1.75	.95	.72	1.27	1.42
Control 2	1.11	2.41	.93	.85	1.27	1.28
Pig #1	48.86	3.5	1.1	.92	1.27	1.46
Pig #2	48.02	2.86	1.33	1.02	1.02	1.44
Pig #3	39.19	3.16	1.03	.71	1.03	.93
Pig #4	1.35	3.08	1.21	1.03	1.03	1.28
Pig #5	.95	3.56	.87	.79	1.06	1.5

*Substitutions, deletions, and insertions in each off-target (OT) site were assessed within a 5-bp window around the predicted Cas9 cleavage site using CRISPResso2. The percentages of sequences were calculated by dividing the read-number of sequences with substitutions, deletions, or insertions by the total read-number of sequences. Ear tissues from two wild-type pigs (control 1 and 2) or gene-edited pigs (pigs #1 to #5) were used as samples.

Piglet #3 died soon after birth. Piglets #2 and #4 suffered from poor health conditions with significant prostration (Supplementary Figure S1A) and exhibited high blood glucose levels 1 day after birth (Supplementary Figure S1B). In accordance with the humane endpoint, early euthanasia was performed on piglets #2 and #4 1 day after birth. Macroscopic necropsy analysis indicated that no abnormalities were detected in major organs, including the pancreas (Supplementary Figure S1C). Next, we performed an inheritance analysis of *INS-*point-mutant pigs. Pig #1 had a leg injury due to an accident; therefore, pig #5 was mated with a WT boar. Eleven F1 piglets were delivered, and eight piglets carried the desired point mutations in *INS*. Three of the eleven F1 piglets carried a monoallelic mutation (1-bp deletion) in *INS*, which was the same mutation seen in pig #5.

**FIGURE 6:**
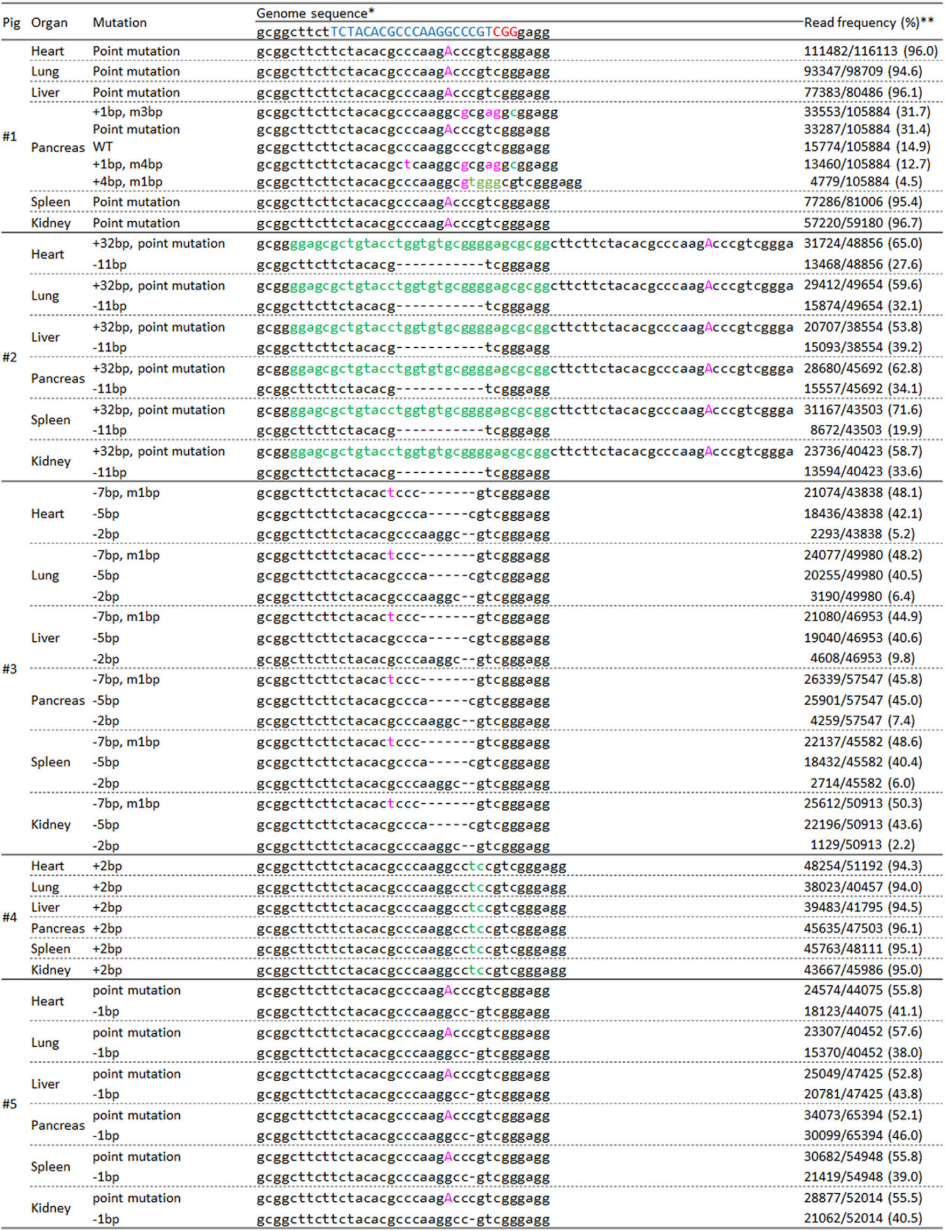
Deep-sequencing analysis of the *INS* target regions in major organs derived from delivered pigs. * The target and PAM sequences of gRNA are indicated in blue and red, respectively. Inserted and modified sequences are represented by green and pink, respectively. ** The frequency was calculated by dividing the number of amplicons by the total number of reads.

Finally, we performed deep sequencing analysis of major organs derived from resulting pigs to evaluate mosaicism accurately (Figure 6). The genotypes (mosaicism) of the major organs in pigs #2 to #5 were similar to that of the ear samples. However, the genotype of pancreas sample in pig #1 showed mosaic mutation although other organs, including the ear revealed biallelic point mutation.

## 4 Discussion

Recently, the generation of point mutations has been widely used to evaluate the functions of enzymes, transcription factors, and signaling molecules. Additionally, the introduction of a desired point mutation in the genomic DNA of an animal model is a novel strategy to mimic intractable human diseases caused by point mutations, such as cancer (Schook et al., 2015), amyotrophic lateral sclerosis (De Giorgio et al., 2019), and Parkinson’s disease (Blandini and Armentero, 2012). Therefore, the introduction of point mutations in pigs has the potential to contribute to human medicine. Previous studies using gene editors demonstrated the generation of point-mutated pigs by SCNT using somatic cells carrying the desired point mutations (Yang et al., 2016; Montag et al., 2018; Li et al., 2020) or the microinjection of gene editors and DNA donors into zygotes (Zhou et al., 2016). In the present study, electroporation successfully enabled the HDR-mediated introduction of a point mutation directly into porcine *in-vitro-*fertilized zygotes and the generation of genetically modified pigs carrying the intended point mutation.

Scr7 temporarily blocks NHEJ and enhances the frequency of HDR, resulting in improvements in the insertion efficiency of DNA fragments at the target loci cleaved by the CRISPR/Cas9 system (Maruyama et al., 2015). In the present study, we simultaneously introduced Scr7 with CRISPR/Cas9 and ssODN into porcine zygotes to improve the efficiency of point mutation introduction. Electroporation of the CRISPR/Cas9 system with 1 μM Scr7 improved the efficiency of point mutation introduction in porcine zygotes, but no apparent effect was observed. We previously evaluated the effect of Scr7 on increasing HDR efficiency targeting another gene, *KRAS*, wherein no improvement was observed in HDR efficiency upon the addition of Scr7 during electroporation. The results of the present and previous studies indicate that the addition of Scr7 during electroporation is limited. Previous studies have reported that 1 μM of Scr7 is an effective concentration for increasing HDR (Maruyama et al., 2015; Ma et al., 2016), whereas some studies demonstrated no significant increase in HDR efficiency with Scr7 treatment (Lee et al., 2016; Song et al., 2016; Park et al., 2017). In the studies that demonstrated Scr7 efficacy, Scr7 was directly injected into zygotes using a glass capillary or introduced into cell lines by culturing for 24 h in Scr7 supplemented medium. The improvement of HDR in embryos cultured with Scr7 after electroporation needs to be evaluated. However, the average percentage of blastocysts from zygotes electroporated with Scr7 was lower than that from zygotes electroporated without Scr7. Another study indicated that higher concentrations of Scr7 reduce cell growth by inhibiting the cell cycle (Maruyama et al., 2015). The cell cycle of zygotes and embryos may also be affected by Scr7, indicating that toxicity should also be evaluated.

Other NHEJ inhibitors and HDR enhancers should be evaluated in porcine zygotes to improve the efficiency of introducing point mutations. RS-1 is a HDR enhancer that enhanced Cas9-and TALEN-mediated knock-in efficiency in rabbit embryos (Song et al., 2016). Furthermore, L755507, a β3-adrenergic receptor agonist, enhanced CRISPR/Cas9-mediated HDR efficiency in human induced pluripotent stem cells (Yu et al., 2015). Using multiple gRNAs, which overlap by at least five base pairs in target sites, enhanced CRISPR/Cas9-mediated knock-in efficiency in mice (Jang et al., 2018). In the present study, Scr7 had no apparent effects on point mutation introduction, but the combination of these strategies with or without Scr7 could improve HDR-mediated gene modification in porcine zygotes.

The rational design of ssODN donors is a key parameter to promote HDR pathway activity. Although ssODNs with longer homology arms can be used to maintain sufficient homology through the prevention of exonuclease degradation, longer ssODNs increase the risk of possible secondary structures, thereby decreasing the number of donors available for HDR (Yang et al., 2013; Howden et al., 2015). Our results demonstrated that the elongation of homology arms decreased HDR-mediated gene modification, which is consistent with the results of previous studies in pigs (Wang et al., 2016; Wittayarat et al., 2021). The adequate homology arm length of ssODNs is an essential parameter to consider.

Moreover, we did not evaluate the optimal concentration of ssODNs. Using the microinjection method, higher concentrations of ssODNs were shown to reduce HDR-mediated gene modification in porcine zygotes (Zhou et al., 2016). In this previous study, the authors considered that higher amounts of ssODNs probably stimulated NHEJ pathways; therefore, the DSBs introduced by the CRISPR/Cas9 system were repaired preferentially by the NHEJ process instead of HDR. Furthermore, the design of ssODNs has the potential to improve the HDR-mediated introduction of point mutations. In the CRISPR/Cas9 system, mismatches in gRNA sequences with DNA-targeting sites around the PAM-proximal region significantly reduced gene editing efficiency (Hsu et al., 2013). The design of ssODNs carrying silent mutations around the 3′end of the gRNA sequence and PAM sequence prevent re-cutting of the target region by CRISPR/Cas9 after HDR events, and result in an improved introduction of point mutations (Zhou et al., 2016). In the present study, we did not design no silent mutations in ssODN. Therefore, re-cutting of the targeting site would reduce the efficiency of a successful introduction of point mutations. Further optimization of ssODNs should be considered to improve HDR efficiency in porcine zygotes and embryos.

CRISPR/Cas-mediated base editor system, which is another approach to introduce a point mutation at a precise position independent of HDR generates mutations at a single-base level (Komor et al., 2016; Gaudelli et al., 2017). Although the currently available base editors have a limited editing window, electroporation-mediated base editing has also been demonstrated in mice zygotes (Kim et al., 2017). Electroporation of base editors into porcine zygotes will be an effective strategy for introducing a single point mutation.

In the present study, we generated five genetically modified pigs carrying various *INS* mutations, including the desired point mutations. Although several factors such as, the target gene, viability of embryos, quality of frozen thawed sperm, and the condition of recipient gilts, affect the resulting litter size; the reason for the low litter size in the present study is unknown. The results of the deep sequencing analysis using ear biopsies and major organs indicated that piglets #2, #3, and #4 carried only frameshift mutations as a result of failure of introducing point mutations. They also exhibited a lethal phenotype with elevated blood glucose levels due to knockout of the *INS* gene. These piglets had macroscopically normal pancreases. Previous histological studies have proven that the pancreas formed in *INS*-deficient pigs completely lack insulin, resulting in a lethal phenotype (Cho et al., 2018). However, our resulting piglet #5 carrying only the desired point mutation and frameshift mutation (−1 bp) did not have this lethal phenotype, which suggests the secretion of functional humanized insulin.

The results of the deep sequencing analysis using ear biopsies indicated mosaicism in piglets #2 and #3. Deep sequencing analysis in major organs also demonstrated similar mosaic genotypes. However, in pig #1, the genotype of pancreas showed mosaicism, including WT alleles, which was not detected in ear samples. In pigs, mosaicism in the germ line is particularly problematic because maintaining resulting pigs until sexual maturity is time- and labor-consuming, and costly. In the present study, mosaicism was also often observed in genetically modified blastocysts (Figure 4). The strategies for reducing mosaicism are critical. High frequency of mosaicism is a major concern for electroporation-mediated gene editing in porcine zygotes (Tanihara et al., 2016; Tanihara et al., 2018; Tanihara et al., 2020b). We showed that careful selection of efficient gRNA is an effective way to reduce mosaicism in genetically modified pigs (Tanihara et al., 2020a; Tanihara et al., 2021). As described above, the improvement of HDR efficiency is also effective to reduce mosaicism by avoiding failure to introduce point mutation. Furthermore, the elevation of Cas9 concentration improves the efficiency of gene editing in mutant blastocysts (Le et al., 2020; Tanihara et al., 2021). These optimizations of the zygotic gene editing system may reduce undesired mosaicism.

However, the improvement of gene-cutting efficiency by CRISPR/Cas9 will affect off-target events. Off-target effects, such as an unexpected DNA cleavage caused by the binding of gene editors to unintended genomic sites, is a major concern in gene editing. In the present study, we designed gRNAs using the COSMID webtool to minimize off-target effects. Modified sequence identified in pigs #1, #2, and #3 was considered a monoallelic single nucleotide polymorphism, because the same modification was detected in one of the WT genomic DNA fragments. Therefore, no off-target events were observed in F0 pigs. In our previous study, the increasing concentration of CRISPR/Cas9 components was effective in increasing gene editing efficiency without off-target events (Le et al., 2020). However, further investigation is crucial, especially for clinical applications in humans that require precise gene modification. To minimize the off-target effects and improve practical gene editing, the latest approaches were developed, including off-target detection by algorithmically-designed software and genome-wide assays, cytosine or adenine base editors, prime editing, Cas9 variants including dCas9 and Cas9 paired nickase, and the chemical modification of gRNA (Naeem et al., 2020). A high-fidelity Cas9 mutant also resulted in high on-target activity while reducing off-target effects in human cells (Vakulskas et al., 2018). However, the potential for off-target events cannot be completely eliminated. We should minimize off-target events by utilizing the latest developed strategies in founder generations, and evaluate possible off-target events of non-mosaic genetically-modified lines prior to clinical application on humans.

In conclusion, we have developed a new approach for generating genetically modified pigs with desired point mutations by electroporating the CRISPR/Cas9 system into zygotes, thereby avoiding the time-consuming and complicated micromanipulation method. This point mutation was successfully inherited in the next F1 generation. Thus, we successfully established an islet donor strain for pig-to-human xenotransplantation through the electroporation-mediated introduction of point mutations into zygotes. However, the efficiency of introducing point mutations is still low. Therefore, efficient practical application to improve HDR-mediated gene modification in porcine zygotes and embryos requires further optimization.

## Data Availability

The original contributions presented in the study are included in the article/Supplementary Material, further inquiries can be directed to the corresponding author.
