# Supplementary material for: Pigs with an INS point mutation derived from zygotes electroporated with CRISPR/Cas9 and ssODN
Source: Front Cell Dev Biol. 2023 Jan 13;11:884340. doi: 10.3389/fcell.2023.884340 (PMC9880039; doi:10.3389/fcell.2023.884340)
Supplement: Supplementary file 1 [file Table1.DOCX]

Supplementary Material

# Supplementary Figures and Tables

## Supplementary Figure


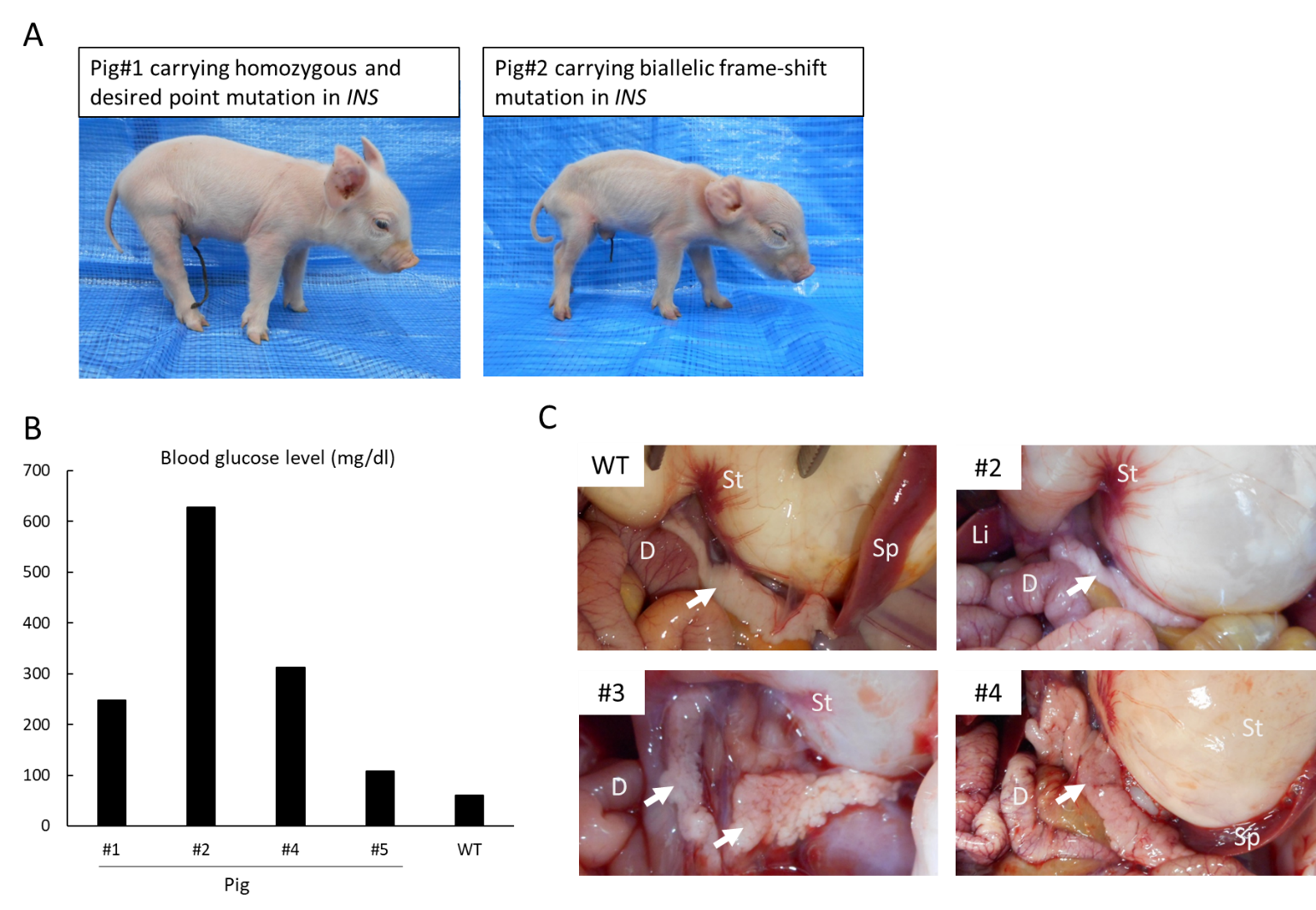


**Supplementary Figure 1. (A)** Macroscopic image of *INS*-deficient piglet #2 with severe health problems and biallelically point-mutated piglet #1 at 1 day after birth. **(B)** Blood glucose levels in *INS* frame-shift mutant and wild-type pigs. **(C)** Macroscopic images of pancreases (arrow) in the *INS* frame-shift mutant and wild-type (WT) piglets. Li, liver; St, stomach; D, duodenum; Sp, spleen.

## Supplementary Tables

| Primer | | Common sequence | |  | Specific sequence |
| --- | --- | --- | --- | --- | --- |
| Forward | | ACACTCTTTCCCTACACGACGCTCTTCCGATCT | |  | AGGACGTGGGCTCCTCTCTC |
| Reverse | | GTGACTGGAGTTCAGACGTGTGCTCTTCCGATCT | |  | CCCAGGGAGTTGGTCACTTT |
|  | |  |  |  |  |

**Table S1. Oligonucleotide sequences used for analysis of the introduced mutations in piglets by deep sequencing**

| Primer | Off-target candidate | Common sequence |  | Specific sequence |
| --- | --- | --- | --- | --- |
| Forward | OT1 | ACACTCTTTCCCTACACGACGCTCTTCCGATCT |  | GTGAGAGAGGCCAAGGAGGA |
|  | OT2 |  |  | CACACAGCAGGCTCTTCAAT |
|  | OT3 |  |  | GAGCCTCACTGTGACCTTCC |
|  | OT4 |  |  | CTCTGATTTGACCCCTAGCC |
|  | OT5 |  |  | TCTGACTCCAGATCCACACC |
|  | OT6 |  |  | CAAAGGAAGCCTCCGAGTCC |
| Reverse | OT1 | GTGACTGGAGTTCAGACGTGTGCTCTTCCGATCT |  | AACTCGATGGTTAAGGGTTCC |
|  | OT2 |  |  | CTCACCCGTTTGGGATTCAG |
|  | OT3 |  |  | ATCTGCCAGTTGGACAGTGC |
|  | OT4 |  |  | AAAGCTCAGCCGTCAACACT |
|  | OT5 |  |  | ATCCGTTTTCACCAATCCTG |
|  | OT6 |  |  | CTGACGCAGCTGGGAAGGTG |

**Table S2. Oligonucleotide sequences used for off-target analysis by deep-sequencing**
